# Different phenotypes of lattice corneal dystrophy type I in patients with 417C>T (R124C) and 1762A>G (H572R) mutations in *TGFBI* (*BIGH3*)

**Published:** 2010-08-13

**Authors:** Pablo Romero, Mauricio Moraga, Luisa Herrera

**Affiliations:** 1 Depto. de Oftalmología, Hospital Clínico “José Joaquín Aguirre,” Universidad de Chile, Santiago, Chile; 2 Servicio de Oftalmología, Hospital del Salvador, Universidad de Chile, Santiago, Chile; 3 Programa de Genética Humana, ICBM, Facultad de Medicina, Universidad de Chile, Santiago, Chile

## Abstract

**Purpose:**

To describe clinical data and to characterize mutations in the transforming growth factor beta-induced (*TGFBI*) gene in patients from three unrelated Chilean families with lattice corneal dystrophy type I (LCDI).

**Methods:**

Snellen acuity tests, anterior segment slit lamp examinations, dilated fundus evaluations, and tonometry were performed for seven patients—five females and two males belonging to three unrelated families—affected with lattice corneal dystrophy Type I. Genomic DNA was also extracted from peripheral leukocytes from the seven patients and four healthy relatives. The 417C>T mutation (R124C) was screened using PCR-RFLP for the seven patients and four healthy relatives. Exons 11, 12, 13, and 14 were sequenced in one patient not carrying the mutation in codon 124. Comparison of phenotype to genotype was performed.

**Results:**

The seven patients studied exhibited LCDI in both eyes, most of which were symmetric. Affected individuals demonstrated progression from central subepithelial needlelike deposits and polymorphic anterior stromal opacities. The age at onset of symptoms varied between six to 15 years old in Family One; the patient in Family Two was five years old and the patient in Family Three was 21 years old. Visual acuity varied from 1.0 to 0.05. Two patients, aged 50 and 45 years, underwent penetrating keratoplasty in both eyes, and two patients, aged 47 and 24 years, underwent penetrating keratoplasty in one eye. The only patient in Family Three exhibited a somewhat distinct phenotype, with yellowish discoloration in the anterior stroma and fewer, but thicker lattice lines than the patients in Families One and Two. Screening for the mutation C>T at the nucleotide position 417 (R124C) in exon 4 in the three families revealed the heterozygous R124C mutation in Families One and Two. In Family Two, the mutation was a de novo mutation, as neither parent was a carrier. Screening by sequencing analysis for mutation in exons 11, 12, 13, and 14 in the affected patient in Family Three revealed a heterozygous A1762G mutation (H572R) in exon 13.

**Conclusions:**

This is the second report of the 417C>T mutation and the first report of 1762 A>G mutation (H572R) in Chilean patients. The H572R mutation identified is associated with a distinct lattice corneal dystrophy type I phenotype.

## Introduction

Lattice corneal dystrophy (LCD) is one of the most common inherited corneal diseases, characterized by the accumulation of amyloid throughout the middle and anterior stroma, forming a network of branching refractile lines. It develops with recurrent corneal erosion and keratoplasty is frequently required [1]. Four distinct subtypes of LCD have been described: types I (OMIM 122200), II (OMIM 105120), III (OMIM 204870), and IIIA (OMIM 608471). Lattice corneal dystrophy type I is an early onset autosomal dominant dystrophy with variable clinical expression [2].

Thus far, few mutations have been described in the transforming growth factor beta-induced (*TGFBI*) gene as causative of LCD type I. The mutation list includes R124C, V505D, L518P, V539D, A546D, P551Q, L569R, H572R, and V625D [3,4]; the mutation C>T at the nucleotide position 417 (R124C) in exon 4 is the most frequent throughout the world [5-16].

The* TGFBI *gene is located in chromosome region 5q31 [5,17] and encodes for transforming growth factor-beta-induced protein ig-h3 (TGFBIp), formerly known as keratoepithelin (KE), an extracellular matrix protein expressed in many tissues and in the corneal epithelium [18,19]. It is a 68 kDa protein, composed of 682 amino acids, and contains four internal repeat domains (FAS) homologous to one another and to fasciclin-1 of *Drosophila* [20]. Transforming growth factor-beta-induced protein ig-h3 is presumably involved in cell adhesion and migration of various cell types, including epithelial cells, fibroblasts, osteoblasts, endothelial cells, and vascular smooth muscle cells [21-23].

Although TGFBIp is ubiquitously expressed, its accumulation in corneal dystrophies is restricted to the cornea, and no other tissues are affected [24]. Neither TGFBIp’s normal function nor the molecular mechanisms underlying the pathogenesis of corneal dystrophy are yet understood. In the cornea, it presumably contributes to the structure of the extracellular matrix.

In this study, we report three unrelated Chilean families with LCDI. Two families carry 417C>T (R124C) and one carries a 1762A>G (H572R) mutation in *TGFBI*. The clinical characteristics of a lattice corneal dystrophy caused by these two mutations are described.

## Methods

### Patients

All examinations were performed according to the tenets of the Declaration of Helsinki and the present study was approved by the ethics committee of the Clinical Hospital of the University of Chile. All patients were informed about the study and gave signed consent. Three index cases were identified during ophthalmic examination at the Clinical Hospital of the University of Chile. After obtaining informed consent, seven affected and four unaffected members from three Chilean families with lattice corneal dystrophy were enrolled. Families One and Two were not related and the last names and family histories suggest Spanish origin. The proband of Family Three was adopted and we do not have any information about his biological family.

### Clinical evaluations

All participants received a detailed clinical examination that included best-corrected visual acuity (BCVA) according to the best line of Snellen acuity, slit lamp biomicroscopy, color cornea photography, pneumatic tonometry (CT 8 Computerized tonometer; Topcon Ltd, Tokyo, Japan), and dilated fundus examination. Autorefractometry measurement and keratometry were performed (model RM-A7000). We considered high myopia when the refractive error was greater than -6.0 diopters.

The LCD type 1 diagnosis was based on clinical examination. The corneal phenotype of all index patients was assessed by slit lamp examination and the review of biomicroscopic photographs by an investigator who did not know the genetic status.

The lesions were considered to be synchronic if the patients perceived the first symptoms in the second eye within a month of perceiving them in the first. All individuals with corneal commitment were considered clinically affected. Patients were classified in degrees of severity according to best-corrected vision, the number of lesions, and corneal commitment (Table 1).

**Table 1:** Clinical features in the affected patients.

** **	** **	** **	** **	** **	**Best-corrected vision**		** **	** **	** **	** **
**Case**	**Age**	**Gender**	**Status**	**Age of onset (years)**	**OD**	**OS**	**Synchronic**	**Symmetry**	**Degree according to best-corrected vision**	**Degree according to corneal commitment**
**Family 1**										
III-1	81	F	Affected	14	0.1	0.05	Yes	Yes	Bad in both eyes	4 in both eyes
IV-10	53	M	Affected	15	0.7	0.5	Yes	Yes	Very Good in the right eye, Good in the left eye	3 in both eyes
IV-13	50	F	Affected	10	0.1	0.2	Yes	Yes	Bad in both eyes	4 in both eyes
V-17	22	F	Affected	15	1.0	1.0	Yes	Yes	Very good in both eyes	1 in both eyes
V-19	27	F	Affected	6	0.8	0.7	Yes	Yes	Very good in both eyes	3 in both eyes
**Family 2**										
II-1	25	F	Affected	5	0.3	0.4	Yes	Yes	Bad in the rigth eye, Intermediate in the left eye	4 in the right eye and 3 in the left eye
**Family 3**										
I-1	52	M	Affected	21	0.3	0.2	Yes	Yes	Bad in both eyes	4 in both eyes

The phenotypic features of seven patients from the three families affected by lattice corneal dystrophy type I are shown. Corneal phenotypes were assessed by slit lamp examination. The lesions were considered to be synchronic if the patients perceived the first symptoms in the second eye within a month after they appeared in the first eye. The degree of severity according to best-corrected vision was classified as: very good (>0,6), good (<=0,6 and >0,4), intermediate (<=0,4 and >0,33), and bad (<=0,33). The degree of severity according to corneal commitment was classified as follows: 1) without lesions, 2) subepithelial, white-grayish opacities, 3) refractile lattice lines, and 4) corneal edema or penetrating keratoplasty. The following abbreviations were used male (M), female (F), right eye (OD), and left eye (OS).

### Molecular genetic analysis

Peripheral blood (5 ml) was collected from seven patients and four unaffected family members and genomic DNA was isolated [25]. The 417C>T (R124C) mutation in exon 4 of *TGFBI* was analyzed in all patients and controls using polymerase chain reaction-restriction fragment length polymorphisms (PCR-RFLP), as previously described [26]. Briefly, genomic DNA was amplified by PCR using the primers TGFBI F1: 5′-CTT TCC CAC ATG CCT CCT CGT-3′ (forward) and TGFBI Rmut: 5′-TCT CAG GCC TCA GCT TCT CCC TGC-3′ (reverse). The PCR amplification was performed as follows: the initial step of denaturation at 95 °C for 5 min was followed by 35 cycles of 1 min each, including denaturation at 95 °C, annealing at 58 °C, elongation at 72 °C, and a final extension at 72 °C for 5 min. The 221 base pair (bp) products obtained were digested using the PstI restriction enzyme (Fermentas, Vilnius, Lithuania) for 3 h at 37 °C. Fragments were resolved by electrophoresis in 3.5% agarose gel, stained with ethidium bromide, and visualized under ultraviolet light. After digestion, the “T” mutant allele consisted of three fragments of 21, 76, and 124 base pairs and the “C” normal allele consisted of two fragments of 97 and 124 base pairs.

Exons 11, 12, 13, and 14 of *TGFBI* were amplified by PCR using the primers and conditions described previously [27]. The primers encompassed the entire exons and short segments of the flanking introns. The PCR amplifications were performed as follows: the initial step of denaturation at 95 °C for 5 min was followed by 35 cycles of 1 min each, including denaturation at 95 °C, annealing at either 58 °C (exon 11, 12, and 14) or 60 °C (exon 13), elongation at 72 °C, and a final extension at 72 °C for 5 min. The PCR products were sent for sequencing at Macrogen® DNA Sequencing Service (Macrogen, Seoul, Korea).

Paternity was confirmed by microsatellite typing of 15 short tandem repeat (STR) loci and Amelogenin using the AmpF/STR Identifiler PCR amplification kit (Applied Biosystems, Foster City, CA) under the recommended conditions.

## Results

### Clinical findings

**Figure 1:**
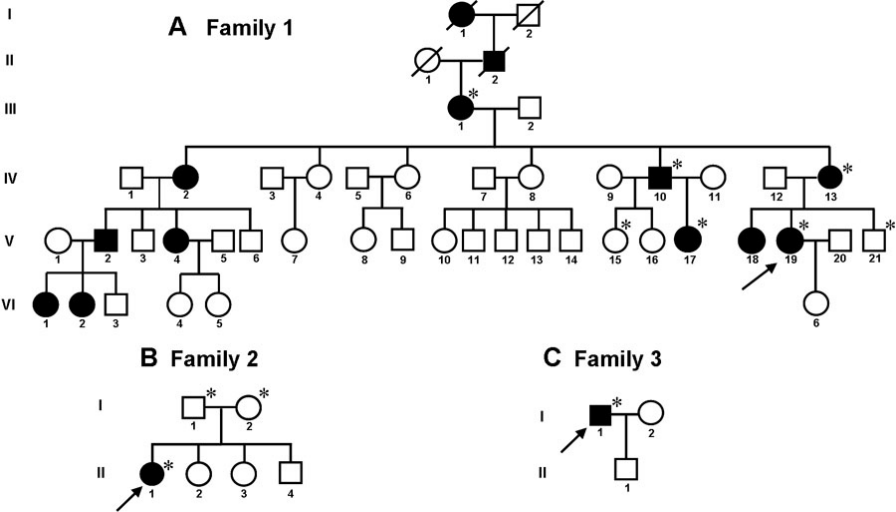
Pedigrees of three families affected by Lattice Corneal Dystrophy type 1. **A**: Pedigree showing six consecutive generations of affected members. **B**: Pedigree showing two generations with just one affected member in the second generation. **C**: Pedigree showing two generations with just one affected member in the first generation. Autosomal dominant transmission of the disease is evident in the first family; the other two families are not informative about the transmission pattern since they are too small and include only one affected individual each. Since LCDI has a dominant transmission and the parents are not affected, the pedigree of Family Two suggests a de novo mutation. The arrows at the lower left of the symbol indicate the probands, open and filled symbols indicate unaffected and affected individuals respectively, squares indicate males, and circles indicate females. Asterisks indicate members of the family who underwent clinical examination and molecular analyses.

Three unrelated index cases were identified during ophthalmic examination at the Clinical Hospital of the University of Chile, Santiago, Chile. The pedigrees of their families were delineated, revealing there were eleven living cases of affected patients in Family One and only one affected member in each of Families Two and Three (Figure 1). We examined some available members of the three families. The phenotypic features of five affected individuals of Family One and the affected individuals of Families Two and Three are summarized in Table 1.

### Family One

The pedigree of Family One is shown in Figure 1A. This was a four-generation family with eleven living affected individuals. Based on an interview with patient III-1, LCD could be traced back over two generations, indicating that there were two dead subjects with a history of LCDI (I-1 and II-2). The presence of LCD type I in the six continuous generations indicates an autosomal dominant transmission. Six relatives of the proband were clinically examined. Four were affected by LCD type I (three females and one male) and two were not affected (one female and one male), as shown in the pedigree (Figure 1A). The phenotypic features of affected family members are summarized in Table 1.

#### Case V-19

**Figure 2:**
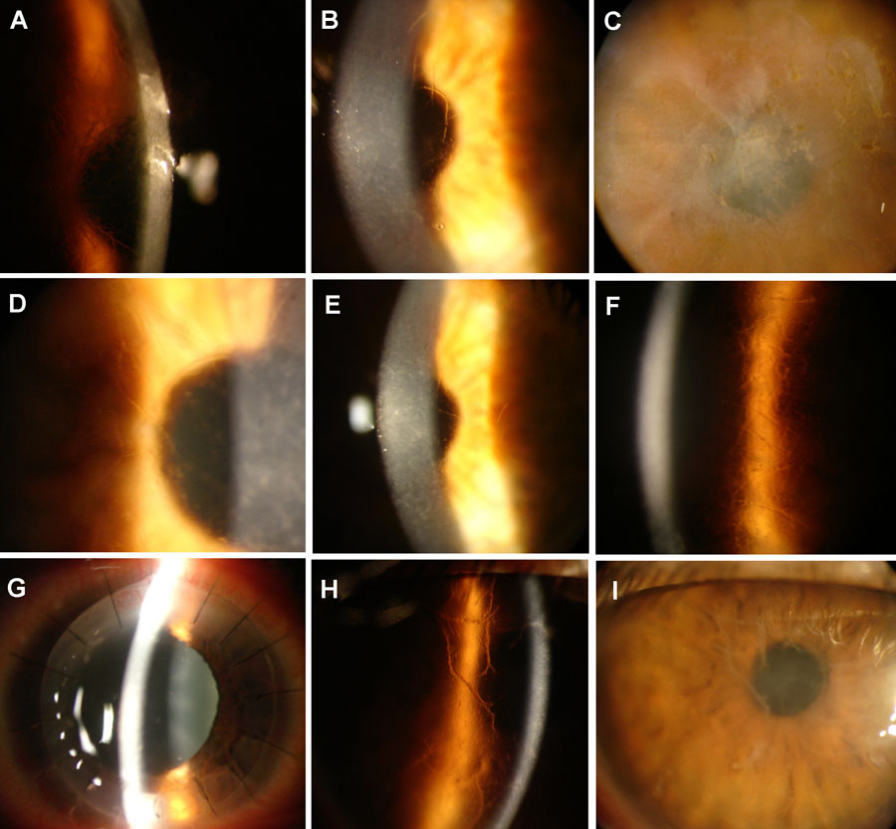
Photographs of the cornea from six individuals examined using slit lamp examination. Slit lamp photographs of patient V-19 of Family One at 27 years of age show opacities in the central stroma and linear forms in the left cornea (**A** and **B**; **A**: OD and **B**: OS). The image of case III-1 shows irregularity of the epithelial surface with subepithelial and anterior stromal scarring in the left eye (**C**). The image of case IV-10 revealed the presence of a network of linear opacities associated with polymorphic anterior stromal opacities in the right eye (**D**). The image of case IV-13 at 50 years of age shows opacities in the central stroma and linear forms in the left cornea (**E**). The image of case II-1 of Family Two at age 25 shows a network of linear opacities associated with other smaller opaque spots and refractile lattice lines in the left eye (**F**) and the right eye shows penetrating keratoplasty with characteristic mydriasis of Urrets-Zavalia syndrome (**G**). The photographs of case I-1 of Family Three at 52 years of age show thick lattice lines and yellowish discoloration in the anterior stroma, resulting in clouding of the central cornea (**H** and **I**).

The proband, a 27-year-old woman, began with episodes of acute ocular pain, redness, and photophobia at six years of age. The frequency and severity of these episodes increased coincident with a gradual deterioration of vision in both eyes. Slit lamp examination showed the presence of large, typical fine branching lattice lines in the anterior stroma of both eyes (Figure 2A,B). A clear area was preserved around the corneal-scleral limbus. She showed a network of linear opacities associated with other smaller opaque spots and refractile lattice lines. No vascularization of the cornea was observed. Central corneal sensation was reduced. Best-corrected vision was 0.8 in the right eye (OD) and 0.7 in the left eye (OS).

#### Case III-1

The proband’s maternal grandmother, an 81-year-old woman, began having symptoms at 14 years of age. She underwent penetrating keratoplasty in the right eye at age 47. Slit lamp examination revealed clinical signs of the recurrence of LCD type 1 in the corneal grafts of the right eye; the corneal graft exhibited a network of linear opacities associated with other smaller opaque spots and refractive lattice lines and diffuse anterior stromal opacity (not shown). Her left eye revealed irregularity of the epithelial surface with subepithelial and anterior stromal scarring, resulting in diffuse clouding of the central cornea (Figure 2C). Peripheral corneal vascularization was observed in both eyes. Visual acuity was 0.1 (OD) and 0.05 (OS).

#### Case IV-10

The proband’s maternal uncle, a 53-year-old man, began with episodes of recurrent corneal erosions at 15 years of age. Slit lamp examination showed the presence of a network of linear opacities associated with other smaller opaque spots and refractile lattice lines in both eyes (OD, Figure 2D). No vascularization of the cornea was observed. He also had high myopia in both eyes. Best-corrected vision was 0.7 in the right eye (OD) and 0.5 in the left eye (OS).

#### Case IV-13

The proband’s mother, a 50-year-old woman, began having episodes of acute ocular pain, redness, and photophobia at 10 years of age. The frequency and severity of these episodes increased coincident with a gradual deterioration of vision in both eyes. At 32 years of age, she was diagnosed with lattice corneal dystrophy. She underwent keratoplasty in her left eye at age 45 and in her right eye at age 47. When she was initially examined at the Clinical Hospital of the University of Chile, slit lamp examination revealed clinical signs of the recurrence of LCD type 1 in the corneal grafts of both eyes. She had bilateral blurred vision; both corneal grafts showed a network of linear opacities associated with other smaller opaque spots and refractive lattice lines and diffuse anterior stromal opacity (Figure 2E). No vascularization of the cornea was observed. Visual acuity was OD: 0.1 and OS: 0.2.

#### Case V-17

The proband’s maternal first cousin, a 22-year-old woman, began with episodes of recurrent corneal erosions at 15 years of age. However, an ophthalmic examination revealed no lesions characteristic of lattice corneal dystrophy, i.e., no typical fine branching lattice lines or vascularization of the cornea were observed. Best-corrected vision was 1.0 in both eyes.

### Family Two

This was a two-generation family with one affected individual in the second generation. The pedigree of this family is shown in Figure 1B.

#### Case II-1

The proband, a 25-year-old women, began with episodes of recurrent corneal erosions at five years of age. She also had high myopia in both eyes. Slit lamp examination showed the presence of elevated subepithelial opacities, fine lattice lines, diffuse “ground glass” haze in the anterior stroma, and corneal grafts (Figure 2F). Best-corrected vision was 0.3 (OD) and 0.4 (OS) (Table1). No vascularization of the cornea was observed. Central corneal sensation was decreased. She underwent penetrating keratoplasty in the right eye at age 24. She was diagnosed with Urrets-Zavalia syndrome in the right eye one month after the surgery (Figure 2G). She did not have a family history of corneal diseases.

#### Case I-1 and case I-2

The proband’s parents, who had no history of recurrent corneal erosions, had a completely normal ophthalmic examination. Best-corrected vision was 1.0 in both eyes for each parent. We also examined three other family members (cases II-2, II-3 y II-4). All were phenotypically normal.

### Family Three

This was a two-generation family with one affected individual in the first generation. The pedigree of this family is shown in Figure 1C.

#### Case I-1

The proband, a 52-year-old man, was adopted and had no knowledge of his parents' ocular status. He began with episodes of recurrent corneal erosions at 21 years of age. He underwent penetrating keratoplasty in the left eye at age 50 and in the right eye at age 51. Slit lamp examination performed before surgery showed the presence of elevated subepithelial opacities, stromal thick lattice lines, diffuse “ground glass” haze, and a yellowish discoloration in the anterior stroma of both eyes (Figure 2H,I). Best-corrected vision was 0.3 (OD) and 0.2 (OS) (Table 1). Vascularization of the cornea and decreased central corneal sensation was observed in both eyes. We also examined his son, who was phenotypically normal.

Dilated fundus examination and tonometry were normal in all members of the three families.

### Molecular genetic analyses

**Figure 3:**
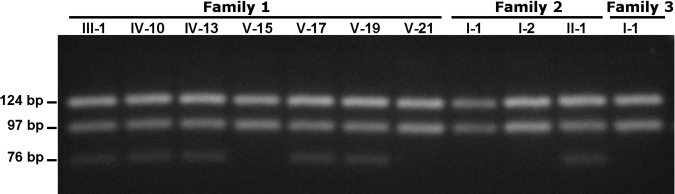
Screening for the 417C>T (R124C) mutation using polymerase chain reaction-restriction fragment length polymorphism. Exon 4 was amplified from individuals III-1, IV-10, IV-13, V-15, V-17, V-19, and V21 of Family One, I-1, I-2, and II-1 of Family Two, and I-1 of Family Three and the PCR product were digested with PstI restriction enzyme. After digestion, the products were analyzed using 3.5% agarose gel electrophoresis. Allele 417C generates two DNA fragments of 124 and 97 base pairs, and allele 417T generates three DNA fragments of 124, 76, and 21 base pairs. The 21 base pair fragment cannot be observed in this kind of agarose gel electrophoresis. Patients III-1, IV-10, IV13, V17, and V-19 of Family One and II-1 of Family Two are heterozygous for the 417C>T mutation, and individuals V-15 and V21 of Family One, I-1 and I-2 of Family Two, and I-1 of Family Three do not carry the mutation.

Exon 4 of *TGFB1* in individuals III-1, IV-10, IV-13, V-15, V-17, V-19, and V-21 of Family One, I-1, I-2, and II-1 of Family Two, and I-1 of Family Three were analyzed using PCR-RFLP and were digested using the PstI restriction enzyme, as previously described [26]. The results indicate that all affected individuals of Family One carry the heterozygous missense mutation 417C>T (R124C) in *TGFBI*. This mutation was not present in healthy controls. The same mutation was detected in individual II-1 of Family Two, but was absent in both parents (Figure 3). Paternity was confirmed, indicating the R124C mutation was a de novo mutation. Individual I-1 of Family Three does not carry that mutation.

**Figure 4:**
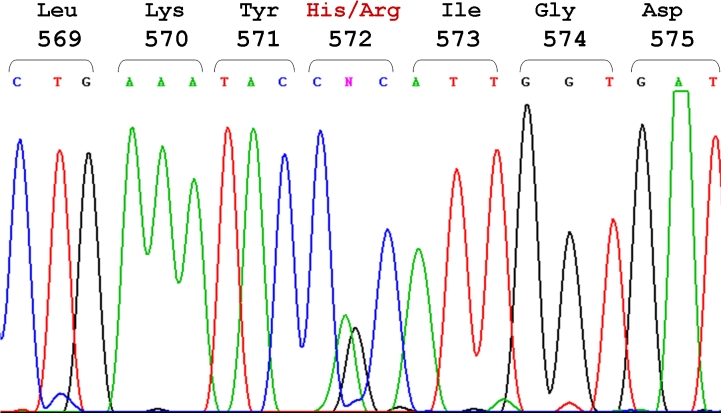
Direct sequencing of exon 13 of the TGFBI gene in the proband of Family Three (individual I-1). The DNA sequence around the codon for histidine 572 (CAC) of the TGFBI gene is presented. The sequence shows a heterozygous, single-base A→G transition at nucleotide 1762, resulting in the replacement of histidine (CAC) with arginine (CGC) (H572R). The codon numbers and the amino acid sequence are indicated at the top of the figure.

Exons 11, 12, 13, and 14 of *TGFBI* of the proband of Family Three were further analyzed by sequencing. The sequence of exon 13 revealed a heterozygous missense mutation 1762A>G that changed histidine to arginine at codon 572 (H572R). This mutation was observed in both direct (Figure 4) and reverse sequences (not shown).

## Discussion

We described the phenotypic characteristics and the mutations in *TGFBI* in seven patients in three families affected with LCDI. We used a combined strategy to identify the mutations. First, we performed a rapid screening based on PCR-RFLP to detect the common 417C>T mutation [26]. When that mutation was not detected, we looked for other putative mutations in exons 11, 12, 13, and 14 of *TGFBI* where other common mutations have been reported [3]. That screening was performed using PCR, followed by direct sequencing of both strands.

The PCR-RFLP analysis revealed the presence of the heterozygous 417C>T mutation in the hotspot of exon 4 in all affected subjects of Families One and Two. This mutation causes the substitution of arginine 124 for cysteine (R124C) in the first FAS domain. Families One and Two were not related, indicating that the mutations at the codon 124 hotspot arose separately and therefore they do not represent a founder effect. Moreover, the parents of the proband of Family Two were both healthy and paternity was proved, demonstrating that the mutation in this family is a de novo mutation and also reaffirming that the 417C>T mutation in Families One and Two did not result from a founder effect. The age at onset of symptoms in individuals of Families One and Two carrying the R124C mutation varied from six to 15 years old. Family One, a relatively extended family, did not exhibit anticipation as we described in a previous report [26].

The 417C>T mutation is the most frequent one reported in *TGFBI*; it has been reported in several ethnic groups throughout the world, including Chile [26]. Consequently, it is not unusual to find a de novo 417C>T mutation in *TGFBI*. In addition, three other different mutations have been described at the same codon, substituting arginine for three different amino acids and generating different phenotypes. These mutations are a transversion 417C>A causing the substitution for serine (R124S) observed in late onset granular corneal dystrophy type I [28], a transition 418G>A causing substitution for histidine (R124H) observed in granular corneal dystrophy type II (Avellino corneal dystrophy) [5], and a transversion 418G>T causing the substitution for leucine (R124L) observed in a form of granular corneal dystrophy type III (Reis-Bucklers corneal dystrophy) [29]. The cause of the high mutation frequency in this codon is unknown. On the other hand, it is interesting that four different amino acids in the same position are associated with different phenotypes. This indicates that Arg124 is critical for normal TGFBIp function. Structural analyses of the FAS1 domain predicted that Arg124 residue would be solvent-exposed and different amino acid substitutions could have very different effects on TGFBI intermolecular contacts and local protein structure [30,31]. In fact, it was demonstrated that R124H substitution abolishes the interaction between TGFBIp and the widely-expressed protein periostin, but R124L and R124C did not. This specific effect leads us to presume that the pathological effects of the substitutions for serine, cysteine, or leucine may be due to the disruption of some other functions [32].

On the other hand, the DNA sequencing of the affected subject of Family Three revealed a heterozygous mutation in exon 13 (A1762G). This mutation changes histidine to arginine at codon 572 (H572R) located in the fourth FAS domain; this has only been reported once, in a Thai family [4]. The age at onset of symptoms of the proband of Family Three was 21. This is the only case observed in that family, thus we cannot be sure that this mutation triggers late onset of the disease, but this at least agrees with the late onset described by Atchaneeyasakul [4]. Additionally, the phenotype of this subject was somewhat different than the phenotype described in subjects with the R124C mutation. For instance, the lattice lines were thicker than the ones observed in patients with the R124C mutation and they were also less crowded. Moreover, in the anterior stroma, we also observed a yellowish discoloration, in agreement with Atchaneeyasakul’s results [4]. In that report, two patients with LCDI exhibited thick yellowish corneal plaque in the anterior corneal stroma. Such yellowish corneal plaque has not been observed in LCD or any other corneal dystrophy [4]. Unfortunately, we did not have corneal buttons available to carry out the histopathologic studies. Since this phenotype is somewhat different than the one observed in other cases of LCD, it is reasonable to believe there may be a genetic cause related to the substitution of histidine for arginine at codon 572 that contributed to the development of this phenotype.

TGFBIp interacts with several extracellular matrix (ECM) proteins, including fibronectin, biglycan, decorin, and several types of collagen [32]. Most of the mutations described in corneal dystrophies, including A1762G (H572R), are located in the fourth FAS domain [3]. Kannabiran and Klintworth [3] suggested that mutations in that conserved domain may alter normal TGFBI protein folding, resulting in the accumulation and deposition of mutant TGFBIp. In fact, TGFBIp deposits have been observed in the cornea [33-36]. It has been postulated that *TGFBI* mutations may modify the secondary and tertiary structures of TGFBIp. This is supported by the observations that TGFBIp may form dimers and tetramers, a characteristic of many proteins capable of amyloid production [37]. The question here is why the substitution of histidine for arginine in position 572 is related to a different color in the anterior stroma and thicker lattice lines than R124C or other mutations [26]. The ability of a specific amino acid substitution to induce amyloid deposition must be related to the location and nature of the substitution, but the role of histidine 572 and the structural effect of its replacement for arginine on TGFBI protein folding are not yet understood and require experimental exploration.

In summary, we report two new families carrying the 417C>T (R124C) mutation and one family with the mutation A1762G (H572R) associated with LCDI. The latter mutation is associated with a distinct phenotype. Further studies are necessary to understand the normal function of TGFBIp and the molecular mechanisms underlying the variegation of phenotypes caused by different mutations.
